# Supplementary material for: Mitogen-like Cerium-Based Nanoparticles Protect Schmidtea mediterranea against Severe Doses of X-rays
Source: Int J Mol Sci. 2023 Jan 8;24(2):1241. doi: 10.3390/ijms24021241 (PMC9864839; doi:10.3390/ijms24021241)
Supplement: Supplementary file 1 [file ijms-24-01241-s001.zip › ijms-2052214-supplementary.pdf]

Table S1. The primers of studied genes in neoblasts subpopulations analyzed by real time (RT) PCR.

Assembly name[a)]	Clone or gene name	Primer F (5'-3')	Primer R (5'-3')
comp1253_c0_seq1	Smed-zfp-1	GCTTCACTCAAAGATCAAATCTACAGA	TTAGAGCAAACTCCACATTTGTATGG
comp2038_c0_seq1	Smed-fgfr-1	CATCCAATAAGTGCGCTTCTCTT	CAGTGGGAACTCCAGAATTGAAA
comp2658_c0_seq1	Smed-soxP-3	CTGTCGATTGGCTGGAAACC	CCGGCCTCGATCAATTACAA
comp3849_c0_seq4	Smed-egr-1	GCCGTGTCCGCTCATCAG	CGGCGAGAAGCCTTATTCCT
comp1153_c0_seq1	Smed-soxP-1	GCTGAGAACGCCTGAGACTATAAAA	TGAATCTAGGAATAACAGCCAATTAGG
comp1412_c0_seq1	Smed-nlk-1	CCCAAGAGGTTGTGACCCAATA	CCACATGTCGACAGCGAATG
comp1749_c0_seq1	Smed-soxP-2	CGATTGATATAATCTGTCATGCTGTTG	CACGCCCTGAATACGACATTG
comp3519_c0_seq6	Smed-fgfr-4	GCCAAAAAACCCACATTTACCA	CTTTGACGAAATCATGCACATTAGA
comp3740_c0_seq1	Smed-inx-13	TTGTTCCAGGGCGTTTCG	CCTCTCCGGCAAAGATTGG
comp4155_c0_seq1	Smed-smad6/7	GACTGACGACAAACTGGAAATCG	CTTTAGCAGCATCCAGTGCAATT
comp9026_c0_seq1	Smed-soxB-1	CACCAGCTTGGCAGAGAGATG	GCATAACCGGACGCCAGAT
comp986_c0_seq1	Smed-pbx-1	CCGCTCAAATAAATTCGGAAGAT	TCCAGTTACACCTTCAGCAATAAGC
comp2057_c0_seq1	Smed-gata456	TGTGTGAACTGTGGAGCTAGCAA	TTGAGAGAACCTGTCGCATTCAT
comp3137_c0_seq1	Smed-hnf4	CGACGCGTTAGACGCATAGAC	GCTTCCTCGATGCAATATCCAT
comp4402_c0_seq1	Smed-prox-1	TCATTTACGAAAGGCCAAACTTATG	TTGTAGAACGTGATATCAGGAAAATAGC

[a)] (Genes of markers neoblasts subpopulations: cyan – the ζ-class, light cyan – the σ-class, yellow – the γ-class)

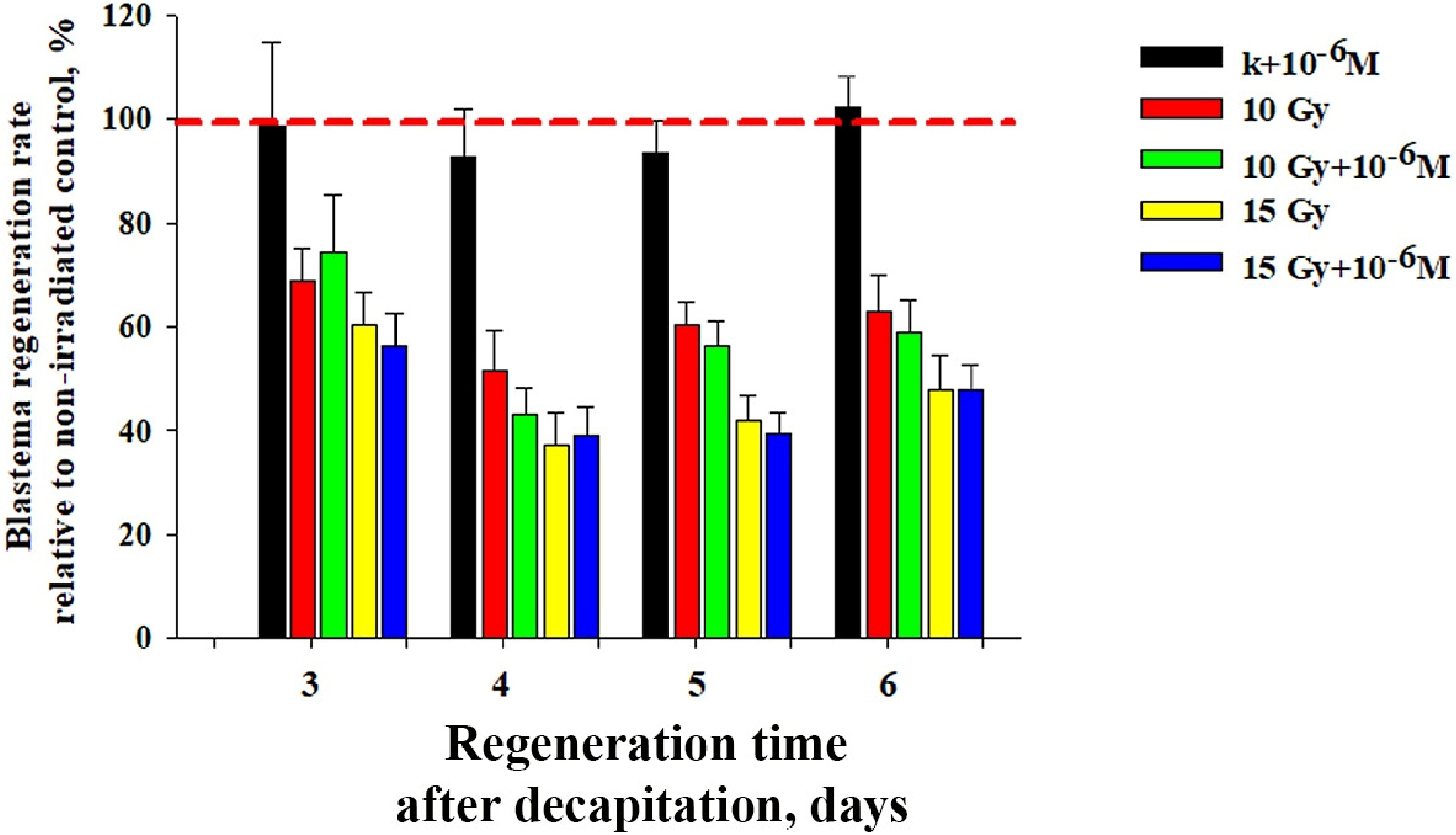

Figure S1. Analysis of the effect of cerium chloride on the rate of regeneration of irradiated planarians. M±SD, n=90.
